# Blood *NCAPH2* Methylation Is Associated With Hippocampal Volume in Subjective Cognitive Decline With Apolipoprotein E ε4 Non-carriers

**DOI:** 10.3389/fnagi.2021.632382

**Published:** 2021-02-02

**Authors:** Ying Chen, Tao-Ran Li, Shu-Wen Hao, Xiao-Ni Wang, Yan-Ning Cai, Ying Han

**Affiliations:** ^1^Department of Neurology, Xuanwu Hospital of Capital Medical University, Beijing, China; ^2^Department of Neurology, Zhejiang Taizhou Municipal Hospital, Taizhou, Zhejiang, China; ^3^Department of Neurobiology, Xuanwu Hospital of Capital Medical University, Beijing, China; ^4^Center of Alzheimer's Disease, Beijing Institute for Brain Disorders, Beijing, China; ^5^National Clinical Research Center for Geriatric Disorders, Beijing, China

**Keywords:** Alzheimer's disease, *NCAPH2*, methylation, subjective cognitive decline, SCD

## Abstract

**Objective:** This study assessed the methylation of peripheral *NCAPH2* in individuals with subjective cognitive decline (SCD), identified its correlation with the hippocampal volume, and explored whether the correlation is influenced by apolipoprotein E ε4 (APOE ε4) status.

**Methods:** Cognitively normal controls (NCs, *n* = 56), individuals with SCD (*n* = 81), and patients with objective cognitive impairment (OCI, *n* = 51) were included from the Sino Longitudinal Study on Cognitive Decline (NCT03370744). All participants completed neuropsychological assessments, blood tests, and structural MRI. *NCAPH2* methylation was compared according to the diagnostic and APOE ε4 status. Partial correlation analysis was conducted to assess the correlations between the hippocampal volume, cognitive tests, and the *NCAPH2* methylation levels.

**Results:** Individuals with SCD and patients with OCI showed significantly lower levels of *NCAPH2* methylation than NCs, which were independent of the APOE ε4 status. The *NCAPH2* methylation levels and the hippocampal volumes were positively correlated in the SCD *APOE* ε4 non-carriers but not in the OCI group. No association was found between the *NCAPH2* methylation levels and the cognitive function.

**Conclusion:** Abnormal changes in blood *NCAPH2* methylation were found to occur in SCD, indicating its potential to be used as a useful peripheral biomarker in the early stage of Alzheimer's disease screening.

## Introduction

Alzheimer's disease (AD) is a progressive and highly debilitating neurodegenerative disorder, accounting for about two-thirds of 50 million people with dementia worldwide (Lane et al., 2018). There is still no therapy to treat the underlying cause of the disease or slow down its progression. The pathogenesis of AD is complex and may involve genetic and environmental factors. A growing body of evidence points to the epigenetic contribution of AD, and both global and gene-specific changes in DNA methylation have been observed in the affected postmortem brain regions (Bakulski et al., 2012; De Jager et al., 2014; Cronin et al., 2017). The search for peripheral blood epigenetic biomarkers of AD is of particular interest because of the unavailability of brain DNA samples until postmortem and the inability to collect longitudinal brain samples to track disease diagnosis (Bakulski et al., 2016).

Recently, two large-scale epigenome-wide association studies (EWAS) have reported the first replicable and robust association of brain methylation and AD pathology in independent cohorts (De Jager et al., 2014; Lunnon et al., 2014). Given the stable changes in DNA methylation levels in older asymptomatic individuals with amyloid pathology, the altered DNA methylation in the brain has been identified as an early feature of preclinical AD. *NCAPH2* is a subunit of the condensin-2 complex involved in chromosome condensation during mitosis and meiosis (Martin et al., 2016). Peripheral blood DNA methylation levels in the *NCAPH2/LMF2* promoter region were significantly decreased in patients with AD and those with amnestic mild cognitive impairment (aMCI) (Kobayashi et al., 2016); therefore, these are considered to be a potentially useful biomarker for the diagnosis of AD. However, while the hypomethylation of *NCAPH2* has been reported in the Japanese population, its presence in the Chinese population remains unclear. Furthermore, whether *NCAPH2* methylation changes across the AD spectrum is still unknown. To date, there have been relatively few studies devoted to the determination of *NCAPH2* methylation patterns in peripheral blood in the early stages of AD.

Subjective cognitive decline (SCD) is characterized by subjective self-perception of worsening cognitive capacity but without any impairment observed in objective evaluations (Jessen et al., 2014; Molinuevo et al., 2017). It has been suggested that SCD precedes MCI and is the preclinical stage of AD (Rabin et al., 2017). Several studies have shown that SCD may be associated with risk factors for dementia, such as the apolipoprotein E (APOE) ε4 allele and the neuropathology of AD (Perrotin et al., 2015; Risacher et al., 2017; Vogel et al., 2017). Thus, recognizing the early characterization of DNA methylation patterns in SCD may help to better understand the role of methylation in the early stages of AD.

Recent research has demonstrated a robust decrease in global DNA methylation in the hippocampus of patients with AD, and this was significantly correlated with amyloid plaque load (Chouliaras et al., 2013). Moreover, a large multisite EWAS revealed DNA methylation in the superior temporal gyrus-mediated associations between blood DNA methylation and the hippocampal volume (Jia et al., 2019). These findings suggest that DNA methylation and the relationship with the hippocampal volume are involved in the pathophysiology of AD. However, whether DNA methylation changes in the blood are correlated with the hippocampal volume in the early stage of AD remain unknown.

Thus, the objectives of this study were (1) to identify cross-sectional differences in peripheral blood *NCAPH2* methylation among patients with SCD and objective cognitive impairment (OCI) relative to normal control (NC) participants and (2) to identify the correlation between the hippocampal volume, the cognitive function, and the *NCAPH2* methylation levels and their interaction with the APOE ε4 status.

## Methods

### Participants

Altogether, 56 NCs, 81 participants with SCD, and 51 participants with OCI (34 MCI and 17 AD) from the Sino Longitudinal Study on Cognitive Decline (SILCODE) project were included in the present research. The SILCODE project is a large, multicenter-based longitudinal observational study in China (ClinicalTrials.gov, NCT 03370744) that is based in Xuanwu Hospital, Capital Medical University, China, and aims to construct a high-precision multimodal model for the ultra-early diagnosis of AD. The study was approved by the institutional review board at Xuanwu Hospital in Capital Medical University, and written informed consent was obtained from all participants.

All 188 individuals fulfilled the inclusion criteria of the SILCODE project (Li et al., 2019). Briefly, SCD is defined using the following criteria (Jessen et al., 2014): (1) self-experienced persistent decline in memory rather than other domains of cognition within the last 5 years; (2) concerns related to SCD and a feeling of worsened performance when compared to others of the same age group; and (3) performance on standardized cognitive tests within age-, gender-, and education-adjusted norms and failure to meet the criteria for MCI or dementia. MCI was diagnosed using the Jak–Bondi approach (Bondi et al., 2014). Impairment in a cognitive domain was defined as having at least two tests >1.0 SD below the age-adjusted normative means or having impaired scores in each of the three sampled cognitive domains (memory, language, and speed/executive functioning). The diagnosis of AD was determined by the published diagnostic criteria (McKhann et al., 1984; Dubois et al., 2007). Individuals with no cognitive complaints and normal performance on the standardized neuropsychological tests were included as controls. The exclusion criteria contained brain trauma or disorder, including clinical stroke, severe psychiatric, and/or severe somatic disease, that may account for symptoms, intellectual disability or other developmental disorders, and other systemic diseases. All participants were assessed using a standardized clinical evaluation protocol comprised of the Mini-Mental State Examination (MMSE), Montreal Cognitive Assessment Basic Version (MoCA-B), Hamilton Depression Scale (HAMD), and Hamilton Anxiety Scale (HAMA).

### Peripheral *NCAPH2* Methylation Measurements

Venous blood samples were collected early in the morning from 6:00 a.m. to 8:00 a.m. Genomic DNA was prepared from leukocytes using the QIAamp DNA Mini Kit (Qiagen, Hilden, Germany), with sodium bisulfite treatment performed as described previously (Ghodsi et al., 2020). Briefly, for the bisulfite reaction (in which cytosine is converted to uracil and 5-methylcytosine remains non-reactive), genomic DNA was initially denatured with 0.3 M NaOH. Sodium metabisulfite solution (pH 5.0) and hydroquinone were then added at final concentrations of 3.0 M and 0.5 mM, respectively. Reaction mixtures were incubated under mineral oil in the dark at 50°C for 16 h. Denatured DNA was purified with Wizard DNA Purification Resin (Promega, Fitchburg, WI, USA), and the reaction terminated by treatment with 0.3 M NaOH at 37°C for 15 min, followed by ethanol precipitation. For bisulfite pyrosequencing analysis, 50 ng of bisulfite-treated genomic DNA was amplified in a DNA Engine Opticon 2 system (MJ Research, Waltham, MA, USA) using a Taq DNA Polymerase kit, hot-start (Takara Bio, Otsu Shiga, Japan). The sequences of the PCR amplification primers, as well as the sequencing primer for *NCAPH2*, were as follows: forward: 5′-GTATTTTTTTGGGAGGGAATAGTAAAATG-3′, reverse: 5′-CCACCTCCCAATTCTTAATAAAA-3′, sequencing: 5′-AGTAAAATGGAGTTAGAATTAGTG-3′, with an amplicon of 187 bp. The reverse primer contained biotin at the 5′ position. The amplification conditions for *NCAPH2* were as follows: 1 cycle of 94°C for 2 min; 50 cycles of 94°C for 20 s, 61°C for 20 s, and 72°C for 20 s; and 1 cycle of 72°C for 5 min. For the pyrosequencing reaction, single-stranded DNA templates were immobilized on Streptavidin Sepharose High Performance beads (GE Healthcare, Uppsala, Sweden) using the PSQ Vacuum Prep Tool and Vacuum Prep Worktable (Qiagen, Hilden, Germany) according to the manufacturer's instructions. Reactions were incubated at 80°C for 2 min and allowed to anneal with the sequencing primers (0.4 mM) at room temperature. Pyrosequencing was performed using PyroMark Gold Reagents (Qiagen) on a PyroMark Vaccum Workstation (Qiagen), according to the manufacturer's instructions.

### APOE Genotyping

Apolipoprotein E was amplified with the following primers: 5′-ACGCGGGCACGGCTGTCCAAGG-3′ (forward) and 5′-GGCGCTCGCGGATGGCGCTGA-3′ (reverse), using the following conditions: 1 cycle of 98°C for 10 s, 35 cycles of 72°C for 5 s, and 1 cycle of 72°C for 5 min. PCR was performed in a final volume of 30 μl, containing 10 pmol of forward and reverse primers and 50 ng of genomic DNA template, using PrimeSTAR HS DNA Polymerase with GC Buffer (Takara Bio), according to the manufacturer's instructions. Then, APOE was genotyped using the standard Sanger sequencing method (Sangon, Shanghai, China).

### MRI Acquisition

All individuals were scanned on an integrated simultaneous 3.0 T TOF PET/MR (Signa PET/MR, GE Healthcare, WI, USA) at Xuanwu Hospital, Capital Medical University, China. The 3D BRAVO T1-weighted sequence was obtained with the following parameters: repetition time (TR)/echo time (TE) = 6.9 ms/2.98 ms, flip angle (FA) = 12°, inversion time (TI) = 450 ms, field of view = 256 × 256 mm^2^, matrix = 256 × 256, slices = 192, slice thickness = 1 mm, no interslice gap, and voxel size = 1 × 1 × 1 mm^3^.

### MRI Processing

Briefly, the entire workflow was as follows: (1) spatially adaptive non-local means denoising, (2) rough inhomogeneity correction, (3) an aligned image into the Montreal Neurological Institute-Hospital (MNI) space, (4) inhomogeneity correction, (5) intensity normalization, (6) non-local intracranial cavity extraction, and (7) subcortical nucleus segmentation. Steps from 1 to 7 were implemented in the volBrain pipeline (http://volbrain.upv.es). The left and right hippocampal volumes were presented as relative values (%), which were measured in relation to the total intracranial volume (Manjon and Coupe, 2016).

### Statistical Analysis

Demographic data and neuropsychological tests were compared by the ANOVA and the chi-squared test for continuous and categorical variables. To compare the *NCAPH2* methylation levels and the hippocampal volumes, a one-way analysis of covariance (ANCOVA) was conducted with age, gender, and years of education as covariates. Bonferroni's multiple comparison tests were conducted for *post-hoc* comparison. We also performed group comparisons among the NC, SCD, MCI, and AD groups. A two-way ANCOVA was used to assess the interactions between the diagnostic status and the APOE ε4 allele on *NCAPH2* methylation controlling for age, gender, and years of education. Then, several partial correlation analyses were conducted with age, gender, and years of education as covariates. First, we assessed the correlations between the hippocampal volumes (left and right side) and the *NCAPH2* methylation levels in the SCD group and further in the SCD APOE ε4 non-carriers and carriers separately to examine whether the correlation differed according to the APOE ε4 status. We also assessed the correlation between the *NCAPH2* methylation levels and the hippocampal volumes in NC and OCI groups. Second, we evaluated the correlation between the *NCAPH2* methylation levels and the cognitive scores (MMSE and MoCA-B) in all individuals and subgroups (APOE ε4 non-carriers and carriers groups). All *p*-values were calculated using two-sided tests, and *p* < 0.05 was considered statistically significant. All analyses were performed using SPSS Statistics (version 24.0, IBM).

## Results

### Demographic Characteristics and Cognitive Function

Table 1 summarizes the demographic and neuropsychological scores for each group. All groups were statistically comparable in terms of sex distribution. The one-way ANOVA showed group differences in age (*F* = 15.79, *p* < 0.001) and years of education (*F* = 8.88, *p* < 0.001). The OCI group was significantly older and less educated than the control and SCD groups. There were significant differences in the APOE ε4 prevalence among groups (χ^2^ = 9.88, *p* = 0.007); patients with SCD and OCI had higher proportions of APOE ε4 carriers (32.1 and 41.2%, respectively) when compared to the controls (14.3%). Patients with OCI showed lower MMSE and MoCA-B scores than controls and individuals with SCD (MMSE: *F* = 53.85, *p* < 0.001; MoCA-B: *F* = 92.62, *p* < 0.001). The SCD and OCI groups scored significantly higher on the HAMD than the controls (*F* = 6.62, *p* = 0.002). Similarly, the HAMA score was higher among the patients with OCI than among the controls (*F* = 4.06, *p* = 0.019), but there was no difference between the SCD and control groups. No significant differences were found in the HAMD or HAMA scores between patients with SCD and patients with OCI (all *p* > 0.1).

**Table 1:** Demographics and clinical characteristics of the subjects.

	**NC (*n =* 56)**	**SCD (*n =* 81)**	**OCI (*n =* 51)**
Age (y)	66.7 ± 5.0	66.4 ± 4.5	71.6 ± 7.31^**^^##^
Male, n (%)	23 (41.1%)	26 (32.1%)	24 (47.1%)
Education (y)	12.4 ± 3.4	12.0 ± 2.9	9.9 ± 3.91^**^^##^
APOE ε4, n (%)	8 (14.3%)	26 (32.1%)1^**^	21 (41.2%)1^**^
MMSE	28.7 ± 1.3	28.7 ± 1.2	22.5 ± 6.5^1^**^^##^^
MOCA-B	26.1 ± 2.0	25.6 ± 2.3	17.6 ± 5.9^1^**^^##^^
HAMD	3.0 ± 3.1	5.0 ± 3.7^*^	5.9 ± 5.51^**^
HAMA	3.3 ± 3.3	4.4 ± 3.6	5.5 ± 4.4^*^

*Results are expressed as mean ± standard deviation. n = number of subjects; NC, normal control; SCD, subjective cognitive decline; OCI, objective cognitive impairment; MMSE, Mini-Mental Status Examination; MoCA-B, Montreal Cognitive Assessment-Basic Version; HAMD, Hamilton Depression Rating Scale; HAMA, Hamilton Anxiety Rating Scale*.

* *p < 0.05*,

** *p < 0.01, compared with NC*;

## *p < 0.01, compared with SCD*.

### Group Differences in the *NCAPH2* Methylation Levels

**Figure 1:**
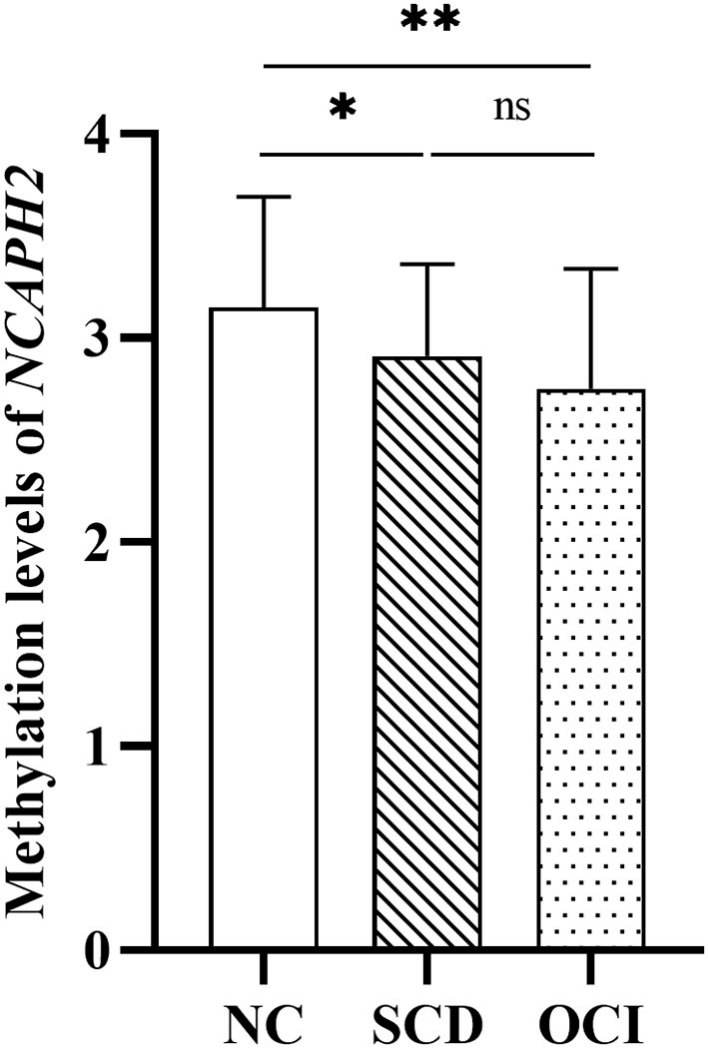
Group differences in the *NCAPH2* methylation levels among the three groups. The analysis was adjusted with age, gender, and years of education. Asterisks indicate *post-hoc* comparisons with respect to controls (Bonferroni corrected). The error bars indicated SDs. *p < 0.05, **p < 0.01; ns, not significant. NC, normal control; SCD, subjective cognitive decline; OCI, objective cognitive impairment.

One-way ANCOVA revealed that the *NCAPH2* methylation levels were significantly different among the three groups (*F* = 6.43, *p* = 0.002) after controlling for age, gender, and years of education. Figure 1 shows that patients with SCD and patients with OCI had significantly lower *NCAPH2* methylation levels than the controls (both *p* < 0.001; Bonferroni corrected), but there was no difference between patients with SCD and patients with OCI (*p* > 0.1; Bonferroni corrected). Further comparison among the four groups showed that the *NCAPH2* methylation levels were lower in patients with MCI and patients with AD than the controls, but there was no difference among patients with SCD, patients with MCI, and patients with AD (Supplementary Figure 1).

The *NCAPH2* methylation levels were significantly low in the APOE ε4 carriers than in the APOE ε4 non-carriers (Supplementary Table 1 and Supplementary Figure 2). Furthermore, no significant additive interactions were observed in *NCAPH2* methylation between the diagnostic groups and the APOE ε4 allele (*F* = 0.41, *p* = 0.665).

### Group Differences in the Hippocampal Volume

**Figure 2:**
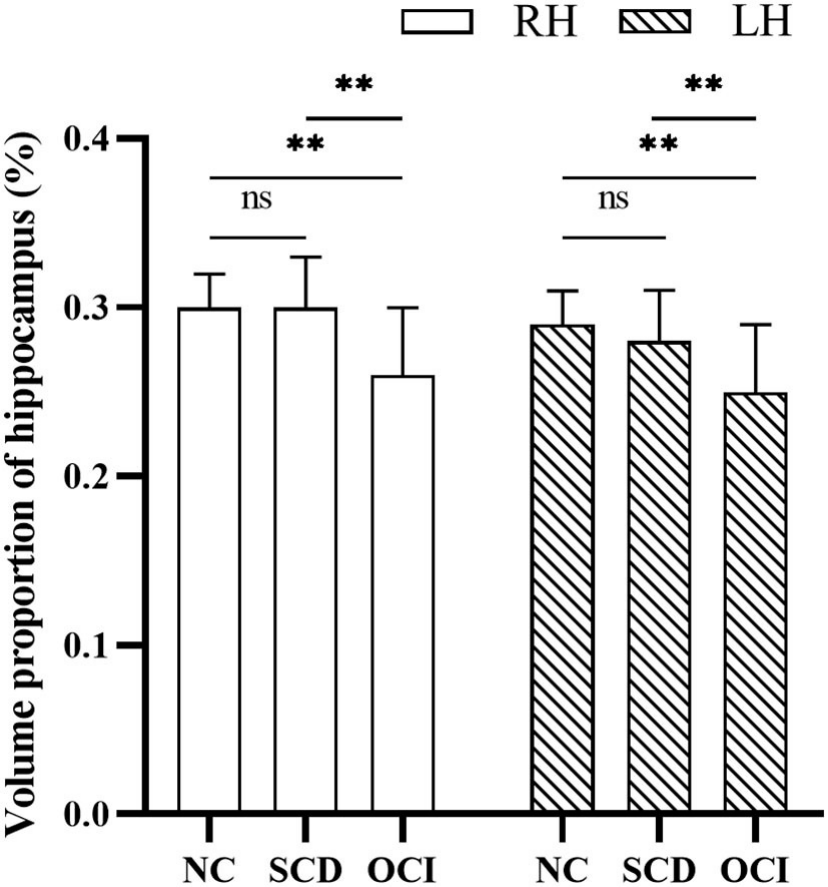
Group differences in the volume proportion of the right and left hippocampus. Results were corrected for multiple comparisons (Bonferroni corrected). **p < 0.01; ns, not significant. NC, normal control; SCD, subjective cognitive decline; OCI, objective cognitive impairment; LH, left hippocampus; RH, right hippocampus.

Figure 2 shows the significant differences in the volume proportion of the bilateral hippocampus among the three groups (right hippocampus: *F* = 27.95, *p* < 0.001; left hippocampus: *F* = 25.61, *p* < 0.001). Compared with the controls and SCD groups, patients with OCI exhibited significant volume loss in the right and left hippocampus (all *p* < 0.001; Bonferroni corrected). However, there were no significant differences between the SCD and control groups (*p* > 0.05; Bonferroni corrected).

### Relationship Between the *NCAPH2* Methylation Levels and the Hippocampal Volume

**Figure 3:**
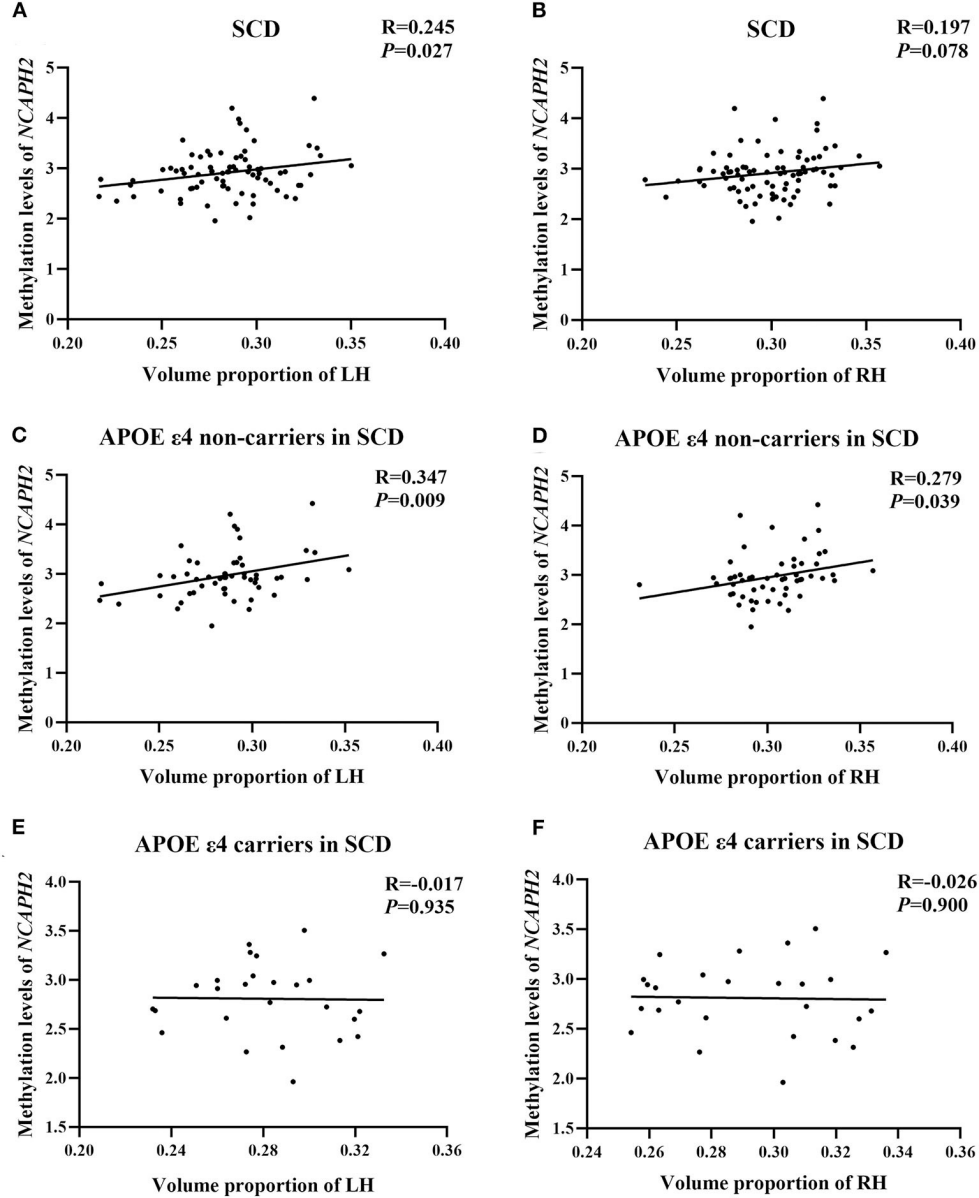
The relationship between the *NCAPH2* methylation levels and the volume proportion of hippocampus in participants with SCD. **(A)** The *NCAPH2* methylation levels were significantly associated with the volume proportion of the LH in participants with SCD; **(B)** The positive correlation was not significant in the RH; Furthermore, the *NCAPH2* methylation levels were significantly associated with the volume proportion of both the LH **(C)** and the RH **(D)** in the APOE ε4 non-carriers. There were no significant associations in the APOE ε4 carriers in the LH **(E)** or the RH **(F)**. SCD, subjective cognitive decline; LH, left hippocampus; RH, right hippocampus.

In the SCD group, there was a significant positive correlation between the *NCAPH2* methylation levels and the volume proportion of the left hippocampus (r = 0.245, *p* = 0.027); however, the positive correlation in the right hippocampus was not significant (r = 0197, *p* = 0.078). Moreover, the positive correlation between the *NCAPH2* methylation levels and the volume proportion of the hippocampus was significant in the APOE ε4 non-carriers in the SCD group (left/right: r = 0.347/0.279, *p* = 0.009/0.039). Nevertheless, there were no significant associations in the APOE ε4 carriers in the SCD group (left/right: r = −0.017/−0.026, *p* = 0.935/0.900) (Figure 3).

The correlations between the *NCAPH2* methylation levels and the volume proportion of the hippocampus in NC and OCI groups were not significant (all *p* > 0.1, Supplementary Table 2).

### Relationship Between the *NCAPH2* Methylation Levels and the Cognitive Scores

There was no correlation between the *NCAPH2* methylation levels and the cognitive scores in all individuals (MMSE: r = 0.077, *p* = 0.304; MoCA-B: r = 0.126, *p* = 0.095, Supplementary Figures 3, 4). Further, subgroup analysis showed no significant correlation in both APOE ε4 carriers and non-carriers groups (all *p* > 0.1, Supplementary Table 3).

## Discussion

In the present study, we found that *NCAPH2* methylation was decreased in the peripheral blood of patients with SCD and patients with OCI compared to the controls. Notably, *NCAPH2* hypomethylation was significantly positively associated with the hippocampal volume in patients with SCD, especially in the APOE ε4 non-carriers. No association was found between the *NCAPH2* methylation levels and the cognitive function.

It has been reported that DNA methylation modifications of several genes are fundamental to the development of AD, which has been considered relevant to AD progression. However, empirical evidence is hard to come by, and the results thus far have been conflicting and controversial. One study has confirmed that higher peripheral methylation levels of brain-derived neurotrophic factor, a member of the neurotrophin family, are associated with a significant AD conversion propensity for patients with MCI (Xie et al., 2017). Other β-amyloid precursor protein (APP)-related genes, such as BACE1, were hypomethylated in the peripheral blood of patients with AD (Marques et al., 2012). Nevertheless, one large case-control study found that there was no difference in the blood DNA methylation levels of *PSEN1* or *BACE1*, both codes for proteins directly involved in the APP cleavage, between patients with AD and the controls, leading to the formation of the β-amyloid (Aβ) (Tannorella et al., 2015). The different methods of methylation analysis and small samples of the varying population may account for divergent findings (Fransquet et al., 2018). The decreased *NCAPH2* methylation in patients with OCI found in our study was consistent with the hypomethylation of the *NCAPH2/LMF2* promoter region reported in a previous Japanese study (Kobayashi et al., 2016). Moreover, the altered methylation pattern was also been found in individuals with SCD, and no difference among patients with SCD, patients with MCI, and patients with AD was observed. Our results indicated that the *NCAPH2* methylation levels decreased during the SCD stage of the disease and reached a plateau at the cognitive impairment stage, suggesting that altered blood *NCAPH2* methylation might be an early feature of AD pathology.

The hippocampus, a brain area critical for learning and memory, is vulnerable to damage at the early stages of AD. In addition, altered neurogenesis in the hippocampus has been suggested as an early critical event in AD due to its relevance for neural plasticity and network maintenance (Mu and Gage, 2011). DNA methylation during neurogenesis has been shown to be responsive to many extrinsic signals, both under normal conditions and during the development of the disease. Our study found that there was a relationship between the *NCAPH2* methylation levels and the hippocampal volume in SCD individuals, but not in patients with MCI and patients with AD. This is not consistent with observations from the previous research, showing a correlation between *NCAPH2/LMF2* methylation and hippocampal atrophy in AD (Shinagawa et al., 2016). A possible explanation for this difference in outcome could be that *NCAPH2* methylation has reached a plateau phase in the early stage of AD, leading to little meaningful variance in methylation levels between patients with cognitive impairment. Thus, a dissociation between *NCAPH2* hypomethylation and the markers of neurodegeneration, including hippocampal atrophy and cognitive impairment, was observed in the late stage of AD. Besides, we observed a significant positive association between the *NCAPH2* methylation levels and the left hippocampal volume rather than the right hippocampus in the SCD group. A previous study of community-dwelling Chinese people showed asymmetry patterns in the hippocampus in SCD, and the correlation between the volume of the right hippocampus and the cognitive function was shown (Yue et al., 2018). Another study reported that SCD with a smaller left hippocampal volume was associated with greater depressive symptomatology (Buckley et al., 2016). Further work is needed to provide a better understanding of the different correlations with hippocampal asymmetry, for instance, in the context of brain function and compensation. Previous studies have shown that epigenetic regulation may be involved in defective T-cell function in *NCAPH2* mutant mice (Gosling et al., 2008). Recent work has identified numerous extravascular CD8+ T-cells in the perivascular space of blood vessels with cerebral amyloid angiopathy in the hippocampi of patients with AD. Furthermore, there was a negative correlation in aMCI and AD between T-cells and cognition (Gate et al., 2020). We hypothesize that dysregulation of *NCAPH2* methylation may lead to an abnormal immune response and finally to hippocampal atrophy. Therefore, a large sample and longitudinal analyses are required to investigate the role of *NCAPH2* methylation, and the correlation with the hippocampus contributes to the pathologic process of AD.

Among the several genes that are considered as risk factors for late-onset AD, the APOE ε4 confers the strongest risk. APOE ε4 isoforms have been shown to affect the disease pathogenesis by regulating Aβ aggregation (Ramanan et al., 2013, 2015) and impairing Aβ clearance in the brain (Castellano et al., 2011). However, it is neither necessary nor sufficient for incident AD; thus, it is of great interest to identify AD risk factors for the APOE ε4-non-carriers population. In this study, we found a positive correlation between *NCAPH2* hypomethylation and smaller hippocampal volume in the APOE ε4 non-carriers of the SCD group but not in the APOE ε4 carriers. Previous studies found that the APOE ε4 carriers have greater hippocampal atrophy than the non-carriers in patients with AD, cognitively normal elderly, and healthy young adults (O'Dwyer et al., 2012; Chang et al., 2019; Dong et al., 2019). This finding further strengthens the idea that AD pathology has multiple factors and suggests that hippocampal atrophy is partly due to DNA methylation in the early stage of the disease. It seems that *NCAPH2* methylation is a useful peripheral biomarker to be used in combination with the analysis of genetic risk alleles to identify the disease pathogenesis, especially in the SCD APOE ε4-non-carriers risk population (Di Francesco et al., 2015).

This study has several limitations. First, the sample size in our study was relatively small. The lack of amyloid biomarkers for the underlying pathology of patients with SCD, patients with MCI, and patients with AD is another limitation of the current study. Further research, especially a follow-up large sample study with biomarkers of AD-type pathology in the preclinical disease stage, is needed to confirm this issue.

Taken together, our results indicated the *NCAPH2* methylation patterns in peripheral blood of individuals with SCD. Moreover, our data revealed the relationship between the *NCAPH2* methylation levels and the hippocampal volume in the APOE ε4-non-carriers of SCD. This information would suggest that changes in blood methylation may be an early indicator of individuals at risk for dementia as well as potential targets for intervention in the early stage of the disease.

## Data Availability Statement

The raw data supporting the conclusions of this article will be made available by the authors, without undue reservation.

## Ethics Statement

Written informed consent was obtained from the individual(s) for the publication of any potentially identifiable images or data included in this article.

## Author Contributions

YH, Y-NC, and X-NW provided data and designed the study. YC and T-RL assembled and analyzed the data, consulted literature, and drafted the manuscript. S-WH reviewed the manuscript. YH and Y-NC critically revised the manuscript for important intellectual content. All authors read and approved the final manuscript.

## Conflict of Interest

The authors declare that the research was conducted in the absence of any commercial or financial relationships that could be construed as a potential conflict of interest.
