# Supplementary material for: Blood NCAPH2 Methylation Is Associated With Hippocampal Volume in Subjective Cognitive Decline With Apolipoprotein E ε4 Non-carriers
Source: Front Aging Neurosci. 2021 Feb 2;13:632382. doi: 10.3389/fnagi.2021.632382 (PMC7884760; doi:10.3389/fnagi.2021.632382)
Supplement: Supplementary file 1 [file Table_1.docx]

**Supplementary Material**

Supplementary Table 1. Demographic characteristics of participants with APOE genotype.

|  | *APOE* ε4 non-carries  (n = 131) | *APOE* ε4 carries  (n = 55) | *p* |
| --- | --- | --- | --- |
| Age (y) | 67.5 ± 5.5 | 69.0 ± 6.9 | 0.101 |
| Male, n (%) | 56 (42.7%) | 17 (30.9%) | 0.131 |
| Education (y) | 11.6 ± 3.4 | 11.5 ± 3.9 | 0.832 |
| MMSE | 27.5 ± 3.8 | 25.8 ± 5.8 | 0.02* |

Results are expressed as mean ± standard deviation. **p*<0.05. APOE ε4, apolipoprotein E ε4; MMSE, Mini-Mental State Examination.

Supplementary Table 2. The correlations between *NCAPH2* methylation levels and hippocampal volume in NC and OCI groups.

|  | RH | | LH | |
| --- | --- | --- | --- | --- |
| Group | r  *p* | | r | *p* |
| NC | 0.067 | 0.632 | 0.080 | 0.568 |
| OCI | 0.110 | 0.505 | 0.135 | 0.427 |

NC, normal control; OCI, objective cognitive impairment; RH, right hippocampus; LH, left hippocampus;

Supplementary Table 3. The correlations between *NCAPH2* methylation levels and cognitive tests in APOE ε4 carriers and non-carriers groups.

|  | APOE ε4 non-carriers | | APOE ε4 carriers | |
| --- | --- | --- | --- | --- |
| Cognitive tests | r | *p* | r | *p* |
| MMSE | 0.056 | 0.540 | 0.036 | 0.803 |
| MoCA-B | 0.078 | 0.396 | 0.096 | 0.507 |

APOE ε4, apolipoprotein E ε4; MMSE, Mini-Mental State Examination; MoCA-B, Montreal Cognitive Assessment Basic Version.

Supplementary Figure 1. Group differences in *NCAPH2* methylation levels among four groups. * *p*<0.05, ** *p*<0.01. ns, not significant. NC, normal control; SCD, subjective cognitive decline; MCI, mild cognitive impairment; AD, Alzheimer’s disease.

Supplementary Figure 2. Group differences in the *NCAPH2* methylation levels of APOE ε4 non-carriers and carriers. ** *p*<0.01. APOE ε4, apolipoprotein E ε4.

Supplementary Figure 3. The relationship between *NCAPH2* methylation levels and MMSE scores. MMSE, Mini-Mental State Examination.

Supplementary Figure 4. The relationship between *NCAPH2* methylation levels and MoCA-B scores. MoCA-B, Montreal Cognitive Assessment Basic Version.
