# Supplementary figures and images for: Blood NCAPH2 Methylation Is Associated With Hippocampal Volume in Subjective Cognitive Decline With Apolipoprotein E ε4 Non-carriers
Source: Front Aging Neurosci. 2021 Feb 2;13:632382. doi: 10.3389/fnagi.2021.632382 (PMC7884760; doi:10.3389/fnagi.2021.632382)

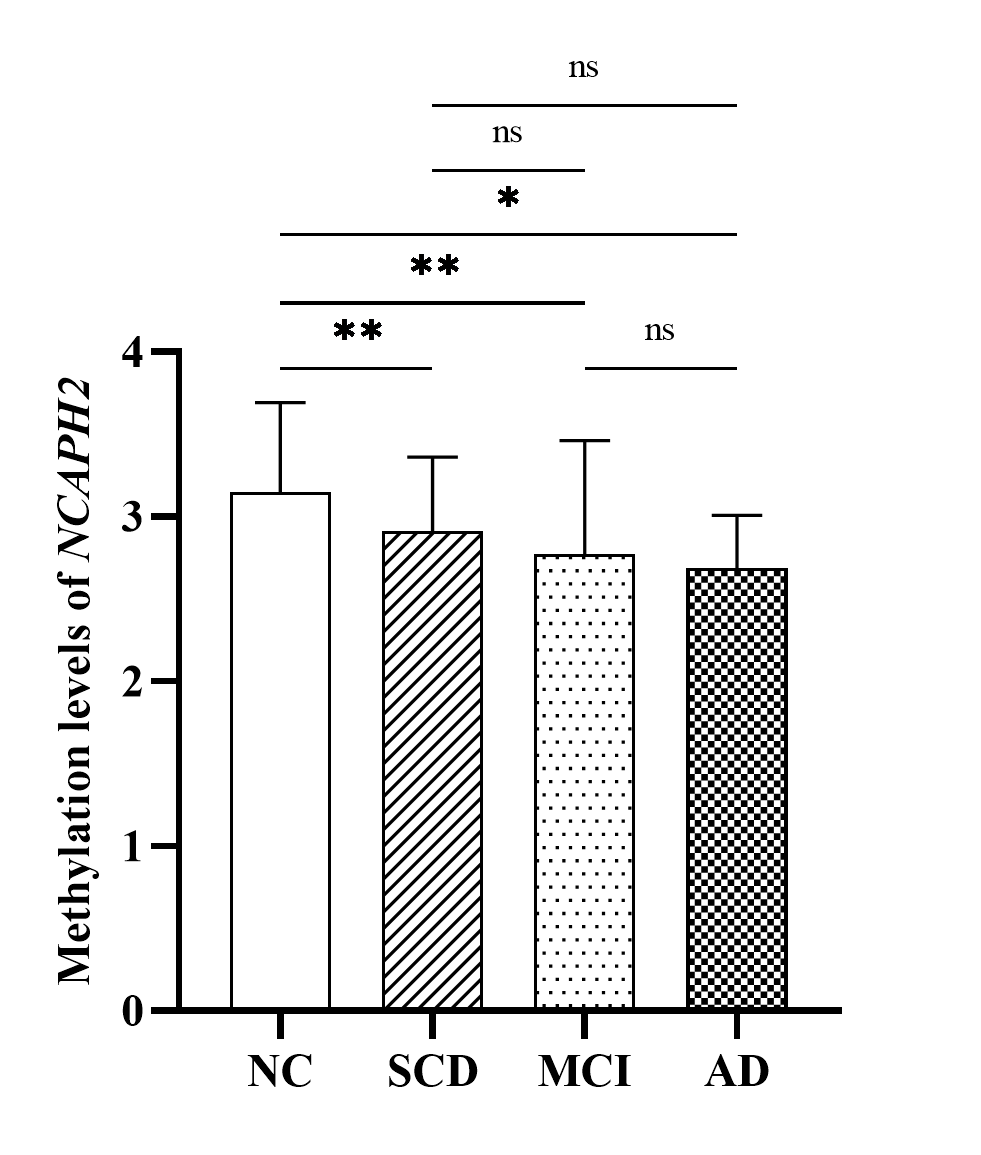

Supplement: Supplementary file 2 [file Image_1.TIF]

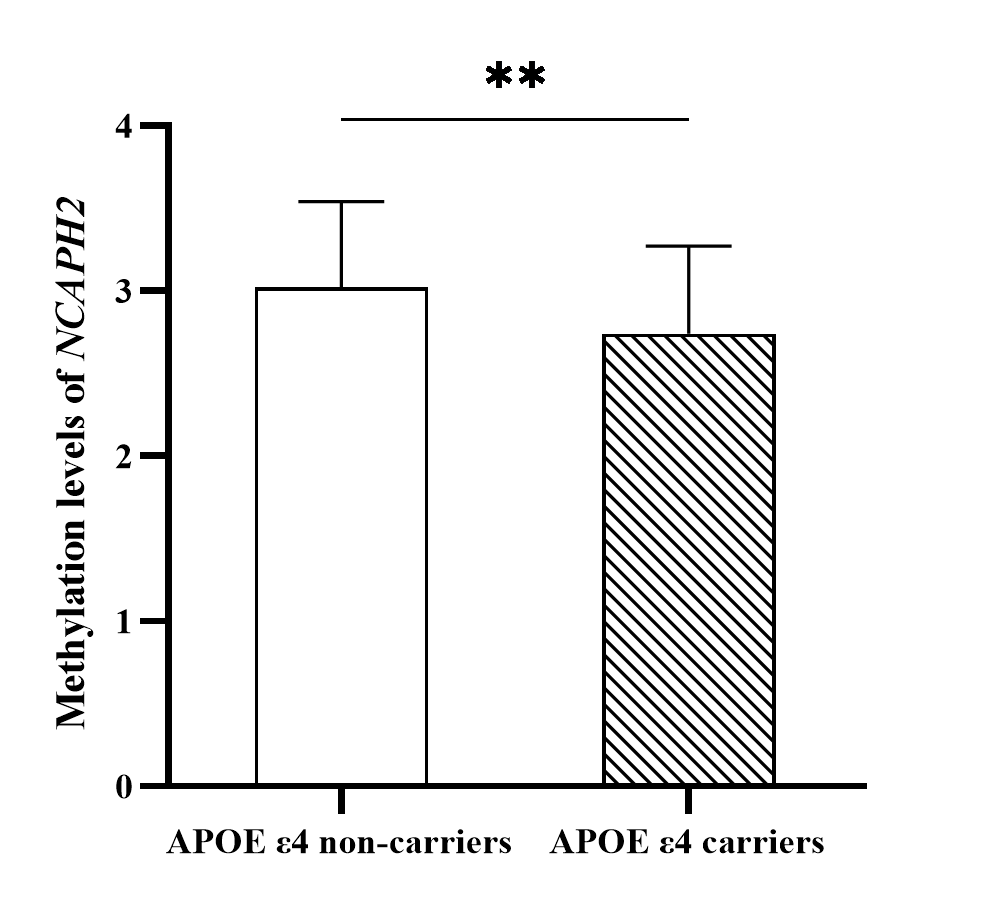

Supplement: Supplementary file 3 [file Image_2.TIF]

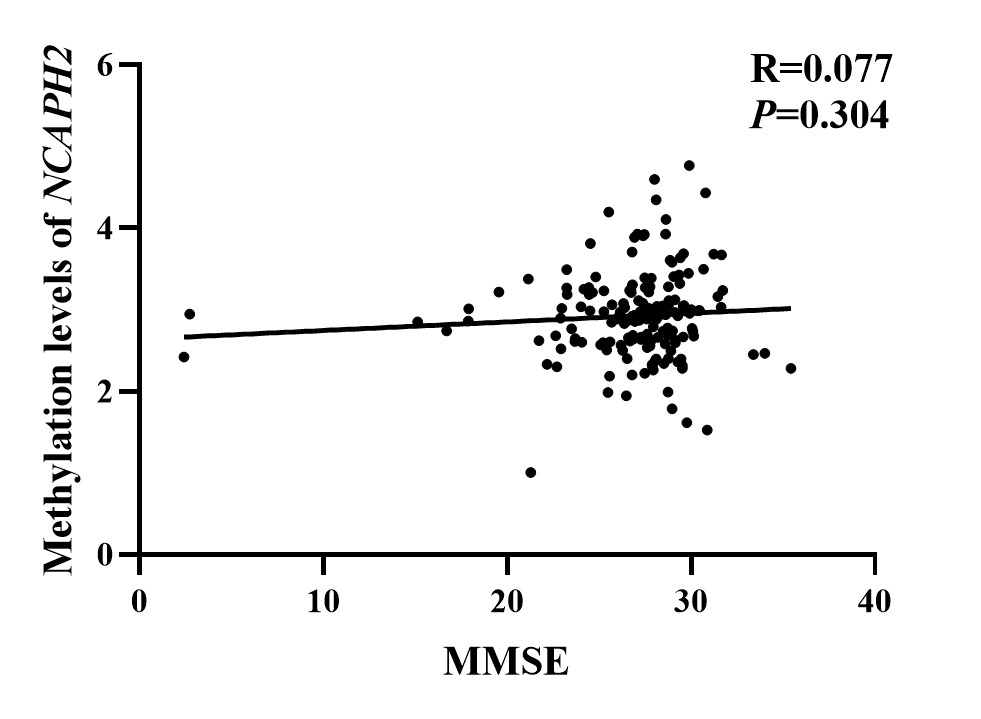

Supplement: Supplementary file 4 [file Image_3.TIF]

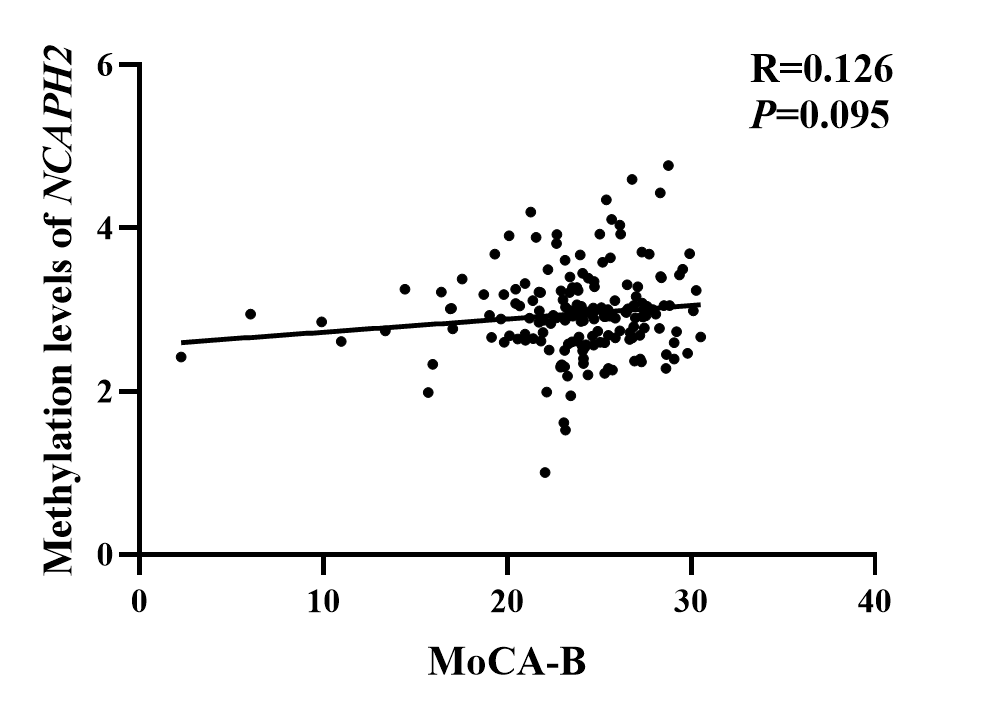

Supplement: Supplementary file 5 [file Image_4.TIF]
